# Simultaneous and recurrent hordeola and chalazia associated with tirzepatide therapy: a case series

**DOI:** 10.1210/jcemcr/luag284

**Published:** 2026-09-17

**Authors:** Andrew Kolker

**Affiliations:** Department of General Ophthalmology, Eye Doctors of Washington, Washington, D.C. 20036, USA

**Keywords:** tirzepatide, GLP-1 receptor agonists, hordeolum, chalazion, meibomian gland dysfunction, ocular adverse effects

## Abstract

Glucagon-like peptide-1 (GLP-1) and dual GLP-1/glucose-dependent insulinotropic polypeptide (GIP) receptor agonists are increasingly prescribed for obesity and type 2 diabetes, but eyelid gland pathology has not previously been reported with these agents. We describe what we believe to be the first reported case series of 2 patients who developed multiple simultaneous internal hordeola after starting tirzepatide, a dual GLP-1/GIP receptor agonist. Patient 1, a 44-year-old woman, developed 6 simultaneous hordeola 4 weeks after dose escalation, 5 of which progressed to chalazia; lesions lessened after tirzepatide discontinuation and worsened upon rechallenge, a positive rechallenge providing strong evidence of causality. Patient 2, a 73-year-old woman with mild pre-existing blepharitis, developed 6 simultaneous hordeola 10 weeks after initiation of her 5 mg dose, 2 of which progressed to chalazia. Both patients lacked classic infectious blepharitis signs despite active lesions, supporting a sterile, obstructive meibomian gland process rather than infection, though both found the recurrences bothersome. These cases suggest a possible, previously undescribed association between tirzepatide and simultaneous or recurrent meibomian gland obstruction. Clinicians should be aware of this potential adverse eyelid effect and pursue conservative ophthalmic management before considering medication changes.

## Introduction

Glucagon-like peptide-1 (GLP-1) receptor agonists and dual GLP-1/glucose-dependent insulinotropic polypeptide (GIP) receptor agonists have become cornerstone therapies for type 2 diabetes mellitus and obesity. Tirzepatide is a first-in-class dual GLP-1 receptor (GLP-1R) and GIP receptor (GIPR) agonist. Reported ocular effects of this drug class include periocular fat atrophy and potential neuro-ophthalmic complications [1-3]. Eyelid gland pathology has not previously been described in association with these agents.

Drug-induced meibomian gland dysfunction (MGD) is an established adverse effect of select systemic medications. Isotretinoin suppresses sebaceous and meibomian gland activity through peroxisome proliferator-activated receptor γ (PPARγ) downregulation, producing chalazia and hordeola [4-6]. EGFR inhibitors have similarly been associated with meibomian gland toxicity and chalazion formation, supporting a broader class of drug-induced eyelid gland disorders distinct from infectious etiologies.

Meibomian glands are specialized sebaceous glands with holocrine secretory function [7]. GLP-1 and GIP receptors are broadly expressed in adipose and dermal tissue, providing a plausible, though not yet directly demonstrated, anatomic basis for a local drug effect at the eyelid margin. GLP-1 RA therapy has been shown to influence sebaceous gland activity, with semaglutide associated with reduced sebum production [8]. We report 2 patients who developed simultaneous internal hordeola and chalazia following initiation of tirzepatide.

## Case presentation

### Case 1

A 44-year-old Black woman presented with swelling of both upper eyelids of 1 week duration. Weight was 142 lb (64.4 kg) and body mass index (BMI) was 25.6. Medical history included discoid lupus (managed without systemic immunosuppression at presentation), attention deficit hyperactivity disorder, and endometriosis. Current medications included methylphenidate extended-release and an oral contraceptive pill. Ocular history was notable for one isolated prior stye without recurrent disease, blepharitis, rosacea, or dry eye disease.

She initiated tirzepatide per the standard titration schedule: 2.5 mg subcutaneous weekly for 4 weeks for obesity, escalated to 5 mg weekly at week 4. Symptoms developed ∼4 weeks after initiation, coinciding with dose escalation to 5 mg.

### Case 2

A 73-year-old White woman presented with swelling of both upper eyelids of 1 week duration. Weight was 156 lb (70.8 kg) and BMI was 28.9. Medical history included breast carcinoma in remission, hypothyroidism, hyperlipidemia, migraines, and depression. Current medications included levothyroxine, bupropion, lifitegrast 5%, and ezetimibe 10 mg orally daily. Ocular history included mild blepharitis, dry eye disease, cataract surgery, and 1 prior isolated stye without concurrent eyelid gland infections.

She initiated tirzepatide per the standard titration schedule: 2.5 mg weekly for 4 weeks, 5 mg weekly for 4 weeks, escalated to 7.5 mg weekly at week 8 for obesity. Symptoms developed 10 weeks after initiation of the 5 mg dose (14 weeks after the first injection), corresponding to 6 weeks after escalation to the 7.5 mg dose.

## Diagnostic assessment

**Figure 1:**
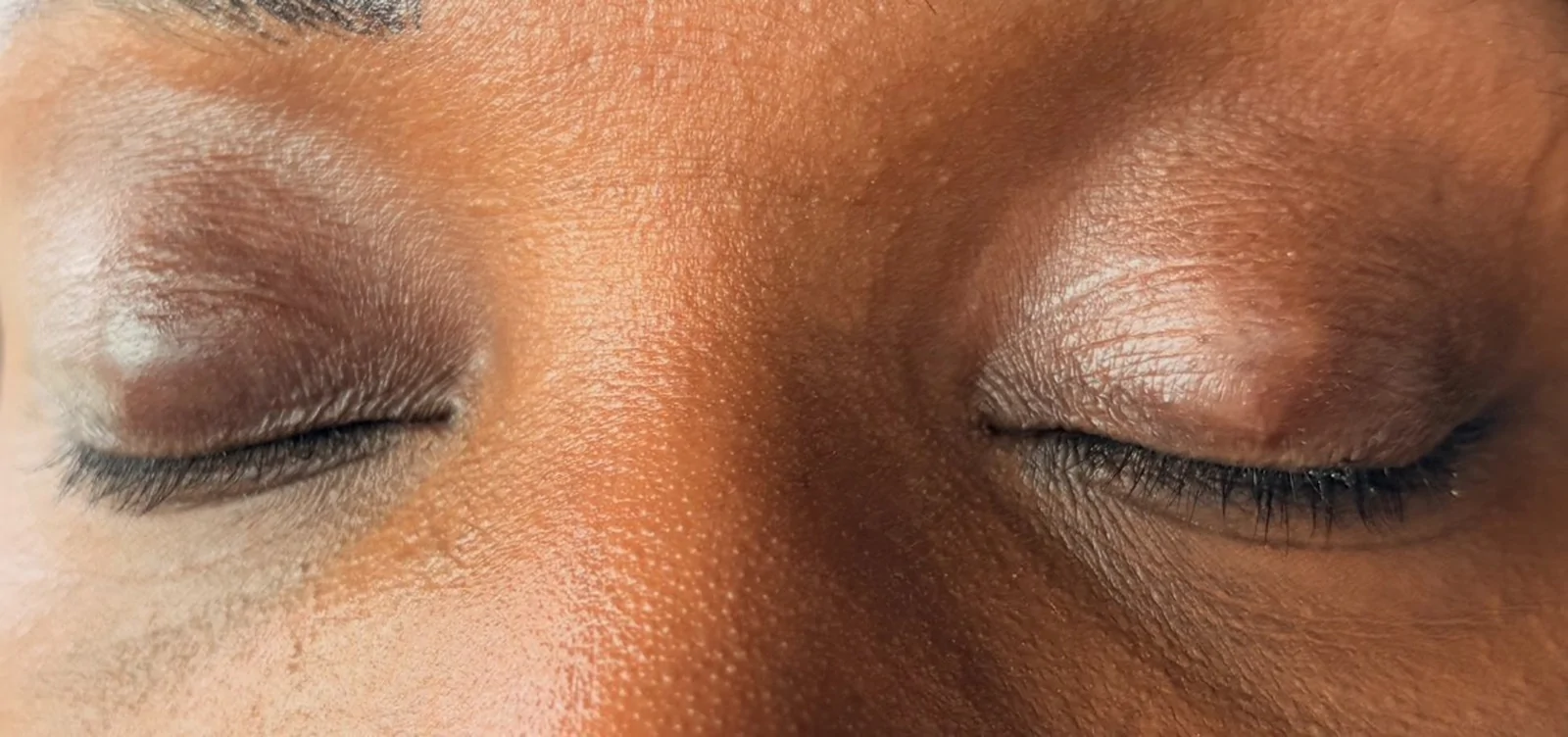
Clinical photograph of patient 1 showing 3 of 6 simultaneous upper eyelid internal hordeola and chalazia. Note the complete absence of lid margin erythema, collarettes, and telangiectasia despite active internal meibomian gland pathology, consistent with sterile obstructive rather than infectious etiology.

Examination demonstrated 6 simultaneous internal hordeola involving both upper eyelids (3 right and 3 left); 5 of 6 progressed to chalazia (Fig. 1). Visual acuity was LogMAR −0.12 bilaterally (Snellen 20/15). Eyelid margin examination demonstrated complete absence of lid margin erythema, collarettes, and telangiectasia, with mild meibomian gland orifice congestion. Meibomian gland expressibility was absent. Tear film was normal. Cultures were not performed.

The complete absence of classic infectious blepharitis signs in the setting of 6 simultaneous active internal meibomian gland lesions is highly atypical for infectious hordeolum and supports a diagnosis of sterile meibomian gland obstruction with chalazion formation [9, 10]. Ocular rosacea was excluded based on the absence of facial flushing, rhinophyma, papulopustular lesions, and prior rosacea diagnosis; new-onset rosacea related to weight loss or systemic stress was also considered but felt unlikely given the absence of any facial or lid margin vascular change. Demodex blepharitis was excluded by the absence of collarettes. Sebaceous carcinoma was excluded clinically. Meibography was not performed at initial presentation given the acute clinical context and is acknowledged as a limitation.

**Figure 2:**
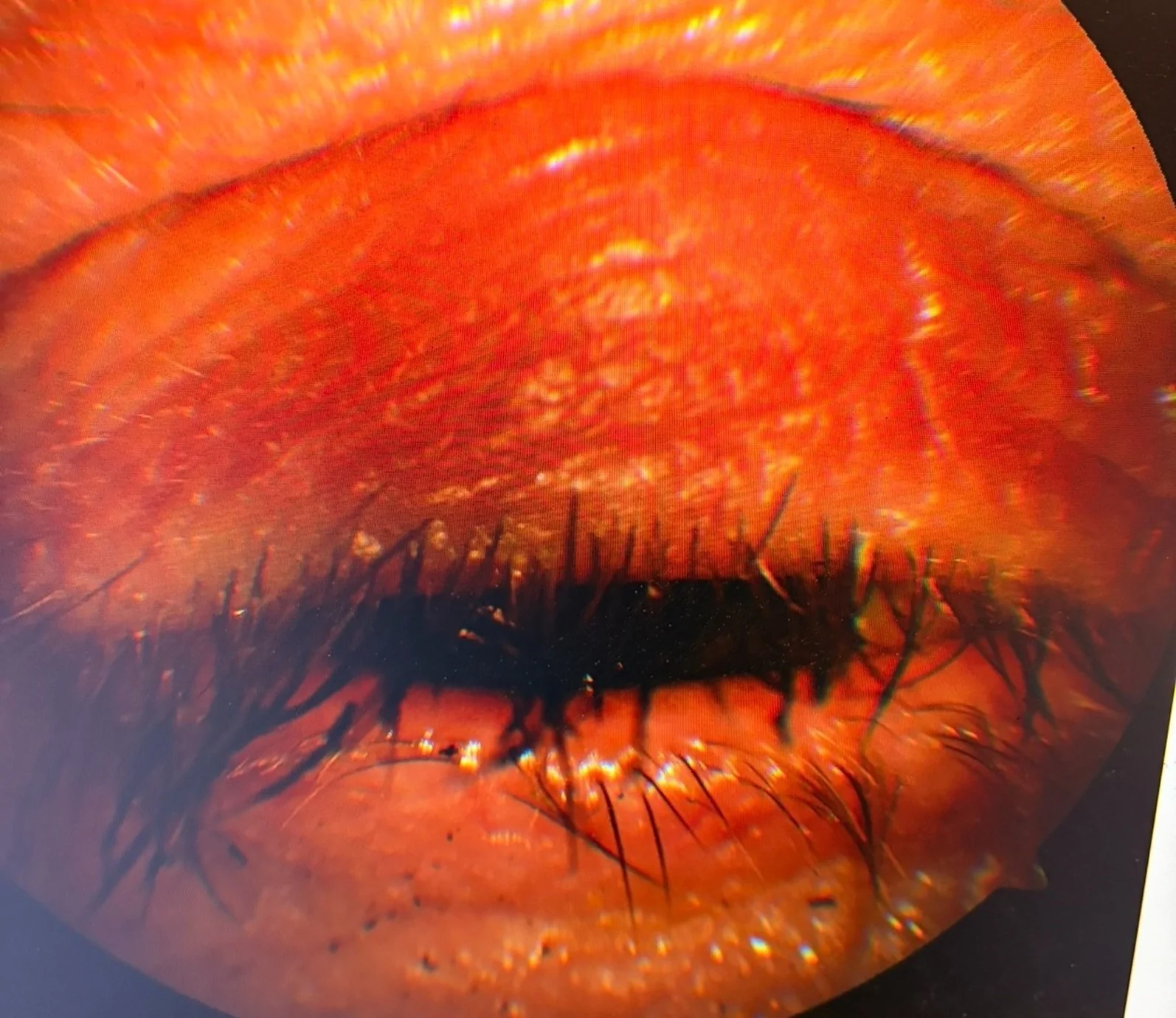
Clinical photograph of patient 2 showing 1 of 6 simultaneous upper eyelid internal hordeola. Note mild lid margin telangiectasia and meibomian gland orifice changes consistent with pre-existing blepharitis.

Examination demonstrated 6 simultaneous internal hordeola involving both upper eyelids (3 right and 3 left); 2 of 6 progressed to chalazia (Fig. 2). Visual acuity was LogMAR 0.00 in the right eye (OD) (Snellen 20/20) and LogMAR 0.10 in the left eye (OS) (Snellen 20/25). Mild lid margin telangiectasia, scurf, and meibomian gland orifice plugging were present, consistent with pre-existing blepharitis; notably, no collarettes were identified. Meibomian gland expressibility was absent. Tear film was reduced. Cultures were not performed.

While pre-existing blepharitis represents a confounding factor, the simultaneous presentation of 6 concurrent hordeola without any prior history of concurrent eyelid gland infections was markedly atypical for her baseline. The absence of purulent discharge, acute conjunctival injection, and collarettes, combined with the hardened nonerythematous lesion morphology, was consistent with sterile obstructive rather than infectious etiology. Ocular rosacea, including new-onset rosacea related to weight loss, and Demodex blepharitis were excluded as in Case 1. Sebaceous carcinoma was excluded clinically.

Patient 1 received a Naranjo Adverse Drug Reaction (ADR) Probability Scale score of 6 (Probable ADR), elevated by the positive rechallenge response. Patient 2 received a Naranjo score of 3 (Possible ADR), reflecting the absence of rechallenge data and the presence of confounding pre-existing blepharitis. The difference in scores reflects the additional causality support provided by the positive rechallenge in Patient 1.

Patient timeline: Patient 1: Week 0 (tirzepatide 2.5 mg initiated); Week 4 (escalated to 5 mg); Weeks 4 and 5 (bilateral upper eyelid swelling onset); Weeks 4-12 (sequential treatment trials without resolution: cephalexin, doxycycline, topical tobramycin/dexamethasone); tirzepatide discontinued; 1-month discontinuation period (lesions lessened); tirzepatide resumed (all remaining chalazia enlarged and became symptomatic).

Patient 2: Week 0 (tirzepatide 2.5 mg initiated); Week 4 (escalated to 5 mg); Week 8 (escalated to 7.5 mg); Week 14 (bilateral upper eyelid swelling onset, 10 weeks after starting the 5 mg dose); Weeks 14-20 (treatment with topical tobramycin/dexamethasone, doxycycline, and intralesional triamcinolone acetonide); Month 5 of follow-up (treated chalazion recurred); 2 additional self-limited hordeola patient-reported between visits. Tirzepatide continued throughout.

## Treatment

### Case 1

Warm compresses and lid hygiene were initiated as first-line conservative measures. Sequential treatment followed: cephalexin 500 mg twice daily for 7 days, followed by oral doxycycline 100 mg daily for 10 days, then topical tobramycin 0.3%/dexamethasone 0.1% twice daily for 14 days; none resulted in lesion resolution. Tirzepatide was not discontinued at the time of initial treatment.

### Case 2

Warm compresses and lid hygiene were initiated as first-line conservative measures. Treatment included topical tobramycin 0.3%/dexamethasone 0.1% twice daily for 14 days, oral doxycycline 100 mg daily for 10 days, and intralesional triamcinolone acetonide 40 mg/mL (0.2 mL) for a persistent chalazion. Tirzepatide was not discontinued at the time of initial treatment.

## Outcome and follow-up

### Case 1

Patient 1 was followed for 2 months. During follow-up with tirzepatide continued, lesions remained stable without new hordeola. Tirzepatide was subsequently discontinued; over 1 month of discontinuation, lesions lessened and no new hordeola developed. Upon resumption of tirzepatide, all remaining chalazia enlarged and became symptomatic with tenderness to palpation, representing a positive rechallenge response consistent with a medication-associated adverse effect. She found the recurrent lesions bothersome but declined to discontinue tirzepatide long-term given its metabolic benefits.

### Case 2

Patient 2 was followed for 6 months with tirzepatide continued throughout. Initial lesions resolved with treatment; however, the intralesional triamcinolone-treated chalazion recurred at 5 months and required retreatment. An additional 2 self-limited hordeola were patient-reported during the interval between visits. Similarly to Patient 1, she found the recurrences bothersome but did not wish to discontinue tirzepatide.

## Discussion

To our knowledge, this represents the first reported case series describing simultaneous and recurrent hordeola and chalazia in association with GLP-1 receptor agonist therapy. The positive rechallenge response in Patient 1 (lesion enlargement upon tirzepatide resumption following partial resolution after 1 month of discontinuation) provides strong pharmacovigilance-grade support for causality.

Two clinical features merit particular emphasis. First, the simultaneous presentation of multiple hordeola in both cases is uncommon without immunodeficiency, systemic inflammatory disease, or significant predisposing lid margin pathology [9-11]. Second, Patient 1 demonstrated complete absence of classic infectious blepharitis signs despite 6 simultaneously active internal meibomian gland lesions, suggesting a sterile obstructive process originating within the meibomian glands rather than conventional infectious hordeolum propagating from external lid margin disease [9, 10].

Both patients met criteria for overweight (Patient 1, BMI 25.6) or obesity (Patient 2, BMI 28.9) at presentation, consistent with the population for whom tirzepatide is prescribed. Patient 1 had no history of hyperlipidemia and was not on antilipidemic therapy. Patient 2 had pre-existing hyperlipidemia managed with ezetimibe, an antilipidemic agent, which was continued unchanged throughout the reported course.

Tirzepatide's dual GLP-1R and GIPR agonism distinguishes it mechanistically from selective GLP-1R agonists such as semaglutide, which does not act on the GIP receptor. GIPR is expressed in adipose tissue and may independently modulate lipid metabolism in sebaceous-type glands through pathways distinct from or synergistic with GLP-1R-mediated sebaceous suppression. Whether GIP receptor activation contributes to meibomian gland secretory dysfunction remains to be determined. Review of the official Summary of product characteristics for both tirzepatide and semaglutide does not list chalazia, hordeola, or MGD among recognized adverse effects, suggesting this may represent a class effect not previously captured in regulatory documentation.

At the molecular level, GLP-1R and GIPR activation influences intracellular lipid metabolism through AMP-activated protein kinase signaling, downregulating lipogenic transcription factors including sterol regulatory element-binding protein-1 (SREBP-1). Meibomian glands depend on active holocrine lipid synthesis for meibum production and may be particularly susceptible to this lipogenic suppression, resulting in reduced meibum output, increased viscosity, and ductal obstruction [7, 8, 12]. Jabin et al demonstrated significant reductions in sebaceous gland output during semaglutide therapy, supporting this pathway [8]. An analogous precedent exists with isotretinoin, which suppresses meibomian gland activity through PPARγ downregulation and is associated with chalazia and hordeola [4-6].

GLP-1 receptor agonist-mediated rapid weight loss is increasingly recognized to cause “GLP-1 face,” including periocular subcutaneous fat atrophy that alters eyelid anatomy and the mechanical efficiency of blinking [13]. This mechanical change may directly obstruct meibomian gland ducts, compounding the proposed secretory dysfunction described above. Patient 1's history of discoid lupus raises the additional consideration of GLP1 RA-mediated immune modulation at the eyelid margin, given emerging evidence of GLP-1 RA effects on interleukin signaling in autoimmune and psoriatic conditions; this warrants further investigation.

Regarding clinical management, when simultaneous or recurrent chalazia develop in a patient receiving GLP-1 receptor agonist therapy, conservative ophthalmic management (warm compresses, lid hygiene, and appropriate topical or systemic therapy) should be initiated promptly. Drug discontinuation should not be undertaken reflexively, as these agents are frequently prescribed for significant medical indications; both patients in this series preferred to continue therapy despite bothersome recurrences. Each case warrants individualized assessment. The positive rechallenge in Patient 1 demonstrates that careful observation following discontinuation and cautious rechallenge, when clinically indicated, can provide valuable pharmacovigilance data.

Limitations include the retrospective design, small sample size, absence of meibography and histopathologic confirmation, absence of systematic weight, HbA1c, eGFR, and lipid panel data, reliance on a week-based rather than exact-date timeline, and inability to exclude all confounding factors. Causality cannot be definitively established. Prospective investigation is warranted.

## Learning points

- Simultaneous and/or recurrent hordeola and chalazia may represent a previously undescribed adverse effect of GLP-1 receptor agonist and dual GLP-1/GIP receptor agonist therapy; a positive rechallenge response provides pharmacovigilance-grade support for this association.
- The absence of classic infectious blepharitis signs (lid margin erythema, collarettes, and telangiectasia) despite multiple simultaneous active internal hordeola is highly atypical and should prompt consideration of a sterile, drug-induced meibomian gland obstructive etiology.
- Patients initiating GLP-1 receptor agonist or dual GLP-1/GIP receptor agonist therapy, particularly those with pre-existing MGD or blepharitis, may benefit from prophylactic eyelid hygiene counseling and warm compress instruction.
- Drug discontinuation should be considered only in refractory cases in consultation with the prescribing endocrinologist or primary care physician; the ophthalmologist should communicate suspected drug-induced eyelid gland pathology and consider pharmacovigilance reporting, as this may represent a class effect not currently listed in official prescribing information.

## Contributors

A.K. identified and managed the clinical cases, conceived the hypothesis, conducted the literature review, and drafted the manuscript. A.K. reviewed and approved the final submitted version and accepts full accountability for all aspects of the work.

## Data Availability

Original data generated and analyzed during this study are included in this published article.
